# Whether the Weather Matters: A Time-Series Analysis of Rainfall and Mechanical Thrombectomy Utilization and Workflow Metrics

**DOI:** 10.7759/cureus.95503

**Published:** 2025-10-27

**Authors:** Hiroyasu Inoue, Masahiro Oomura, Yusuke Nishikawa, Yoshitaka Nakamura, Atsuhito Taishaku, Tomoyasu Yamanaka, Yuta Madokoro, Teppei Fujioka, Mitsuhito Mase, Noriyuki Matsukawa

**Affiliations:** 1 Neurology, Nagoya City University Graduate School of Medical Sciences, Nagoya, JPN; 2 Neurosurgery, Nagoya City University Graduate School of Medical Sciences, Nagoya, JPN

**Keywords:** ais (acute ischemic stroke), door-to-puncture time, mechanical thrombectomy (mt), precipitation, weather conditions

## Abstract

Background and purpose

Whether weather affects the real-world utilization and workflow of mechanical thrombectomy (MT) remains uncertain. We evaluated the association between daily precipitation and MT utilization and explored differences in workflow metrics.

Methods

We conducted a single-center, retrospective time-series study at Nagoya City University Hospital in Nagoya, Japan, over nine years, linking daily meteorological data from the national weather service to consecutive MT procedures. The primary outcome was the difference in daily MT occurrence between rainy (≥5 mm precipitation) and non-rainy days. Secondary analyses assessed stroke etiology strata, the relationship between precipitation amount and MT occurrence, and differences in door-to-puncture (D2P) times overall and during out-of-hours periods.

Results

Across the study period, 134 MT procedures were performed. The proportion of days on which at least one MT was performed was significantly higher on rainy days compared to non-rainy days (5.7% vs. 3.6%; odds ratio 1.63, 95% CI 1.07-2.42; p = 0.02), with a stronger signal for embolic strokes. A positive dose-response pattern was observed between precipitation amount and MT occurrence. Median D2P was shorter on rainy than on non-rainy days (101 vs. 131 minutes; p = 0.047), with the largest difference during out-of-hours (99.5 vs. 142.0 minutes; p = 0.009). Patient outcomes did not differ significantly between rainy and non-rainy days.

Conclusions

Rainy weather was associated with higher MT utilization and shorter D2P, particularly outside regular hours. These findings frame weather as a pragmatic signal for thrombectomy service demand and workflow performance, with potential implications for demand forecasting, on-call staffing, and protocol readiness. Prospective, multicenter studies with formal time-series adjustments are warranted to confirm generalizability and clarify mechanisms.

## Introduction

A common clinical anecdote suggests that the incidence of mechanical thrombectomy (MT) for stroke varies with periods of high and low activity, indicating that meteorological conditions may be an underlying factor. Previous studies on stroke onset and weather have yielded inconsistent results for factors such as temperature and atmospheric pressure [1-7], with limited research on precipitation.

These conflicting findings may be attributed to methodological differences and difficulty in determining precise stroke onset times from hospital databases [8]. Cases of acute ischemic stroke (AIS) due to large vessel occlusion (LVO) requiring MT offer a distinct advantage, as their acute presentation allows for a more accurate assessment of onset time, overcoming a key limitation of prior research.

Therefore, to address a significant gap in the literature, this study aimed to investigate the association between precipitation and MT frequency for LVO. We also examined its relation to specific stroke subtypes and explored the unexpected and clinically relevant effect of rainy weather on treatment workflow efficiency, such as door-to-puncture (D2P) times.

## Materials and methods

Study setting and design

We performed a single-center, retrospective, time-series observational study at Nagoya City University Hospital, a comprehensive stroke center in Nagoya, Japan. Consecutive adult patients who underwent MT for AIS due to LVO were identified from electronic health records. The study period spanned July 1, 2015 through June 30, 2024. Interhospital transfers and in-hospital strokes were excluded to minimize heterogeneity in pre-hospital timelines and eligibility pathways.

Data sources and linkage

Daily meteorological data (precipitation, temperature, relative humidity, and sea-level pressure; maximum/mean/minimum values and daily totals, as available) were obtained from the national weather service and linked to the hospital calendar at the day level in Japan Standard Time (00:00-23:59). Clinical variables were abstracted from medical records, including demographics, vascular occlusion site, stroke etiology, treatment timelines (emergency medical services (EMS) response, D2P, procedure time), angiographic results (modified thrombolysis in cerebral infarction: mTICI), and outcomes at discharge and 90 days.

Exposure definitions and operational time windows

The primary exposure was daily precipitation. A “rainy day” was prespecified as ≥5 mm of precipitation based on preliminary exploration showing that a 1-mm threshold did not discriminate MT occurrence. For descriptive trends, precipitation was also grouped into ordered categories (e.g., 0-4.9, 5-14.9, ≥15 mm). “Out-of-hours” was defined a priori as time outside regular weekday clinical hours (approximately 08:30-17:00) and all weekends/holidays, according to the institutional on-call schedule.

**Figure 1:**
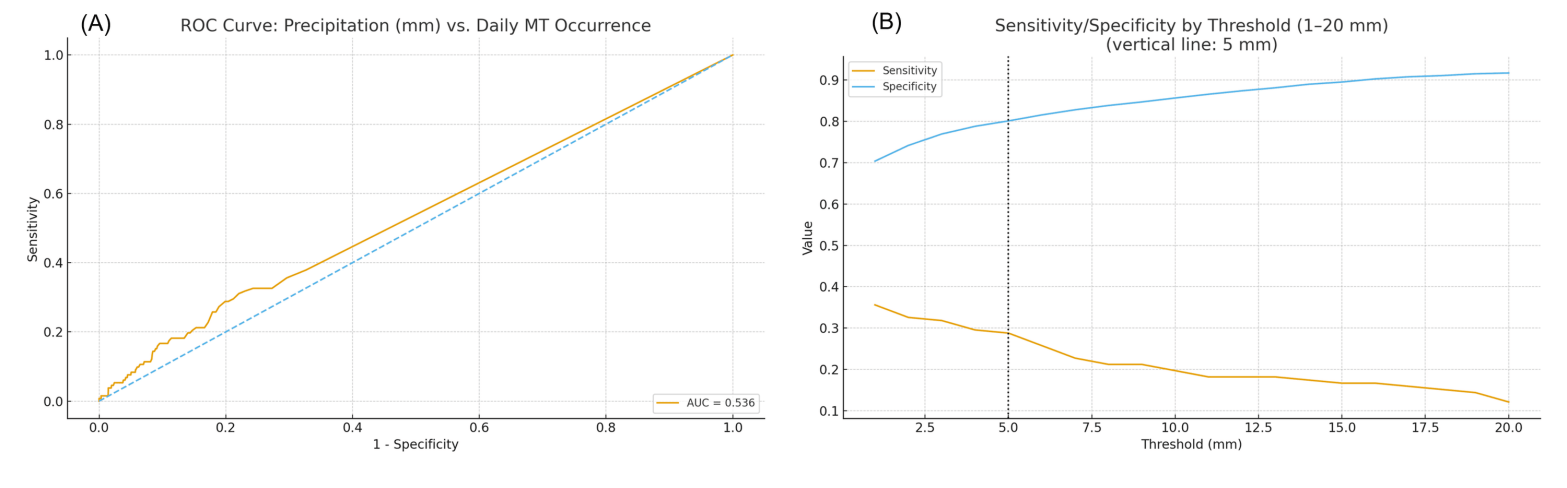
Threshold exploration for defining a “rainy day” cutoff (A) ROC curve for daily precipitation predicting the occurrence of at least one MT per day (AUC = 0.536; 95% CI 0.490–0.581). (B) Sensitivity and specificity across candidate thresholds (1–20 mm); the vertical line marks 5 mm. A shallow plateau is seen around 3–6 mm, with 5 mm providing the best trade-off (sensitivity 0.288; specificity 0.801; Youden’s J 0.089).

Threshold pre-specification and exploratory check. Before defining a “rainy day,” we conducted a brief exploratory check. Daily total precipitation (mm) was treated as a continuous exposure and the occurrence of at least one MT per day as the binary outcome. The ROC analysis showed AUC = 0.536 (95% CI 0.490-0.581). We then scanned candidate cutoffs from 1 to 20 mm, calculating sensitivity, specificity, and Youden’s J (= sensitivity + specificity − 1). The curve exhibited a shallow plateau with a local maximum around 3-6 mm; 5 mm provided the best overall balance (sensitivity 0.288; specificity 0.801; Youden’s J 0.089) and was retained as the prespecified, meteorologically interpretable threshold for the primary binary definition (Fig. 1).

Outcomes

The primary outcome was the daily occurrence of MT (yes/no) on rainy versus non-rainy days. Secondary outcomes were as follows: (i) MT occurrence stratified by stroke etiology (embolic vs. non-embolic); (ii) dose-response pattern between total daily precipitation and MT occurrence; and (iii) comparison of workflow metrics (EMS response time, D2P, and procedure time) and clinical outcomes between rainy and non-rainy days.

Clinical scales and procedural definitions

Neurological deficit was quantified using the NIH Stroke Scale (NIHSS); global disability using the modified Rankin Scale (mRS); early ischemic change using ASPECTS (CT and DWI), including PC-ASPECTS when posterior circulation was involved; and angiographic reperfusion using mTICI grades. Standard scales were applied according to original definitions (NIHSS [9], mRS [10], ASPECTS [11,12], PC-ASPECTS [13], and angiographic mTICI [14]). Licensing/permission details are provided in the table footnotes, where applicable.

Statistical analysis

Analyses were performed at the day level unless otherwise noted. We summarized continuous variables as median (interquartile range) and categorical variables as counts (percentages).

Primary Comparison (Rainy vs. Non-rainy Days)

We used Fisher’s exact test (two-sided) for daily MT occurrence and reported odds ratios (ORs) with 95% confidence intervals (CIs) from logistic regression.

Dose Response

Total daily precipitation was entered as a continuous predictor in logistic models; we additionally displayed ordered categories for descriptive purposes.

Multiple Procedures per Day

To account for days with >1 MT, we fit Poisson regression with a log link and reported incidence rate ratios (IRRs) with 95% CIs; dispersion was examined, and results were compared with the primary specification.

Covariate Specification and Multivariable Modeling

For the multivariable logistic regression of daily MT occurrence, we considered meteorologic covariates a priori. Because mean relative humidity was strongly correlated with precipitation, it was excluded to avoid collinearity. The final model therefore included daily precipitation (primary exposure), minimum temperature, mean sea-level pressure, and maximum instantaneous wind speed. Variance inflation factors (VIFs) were all <2.5 (precipitation 1.15, minimum temperature 1.73, mean sea-level pressure 2.04, maximum wind speed 1.22), indicating no serious multicollinearity.

Group Comparisons for Patient-Level Metrics (e.g.,D2P)

Mann-Whitney U tests (reporting U statistics) were used for continuous variables; Fisher’s exact tests for categorical variables (reporting exact p). Missing data were <1% and were handled by single imputation (median for continuous, mode for categorical). Two-sided p<0.05 was considered statistically significant. Analyses were performed using EZR (Easy R), a graphical interface for R (R Foundation for Statistical Computing).

Ethics

The study complied with the Declaration of Helsinki and was approved by the Institutional Ethics Committee of Nagoya City University Hospital (approval No. 60-24-0133). The requirement for individual informed consent was waived in favor of an opt-out on the institutional website.

## Results

We performed 134 MT procedures over 3,288 days. Rainy days (≥5 mm precipitation) accounted for 20.3% of the days but 29.1% of the MT procedures.

MT frequency and precipitation

**Figure 2:**
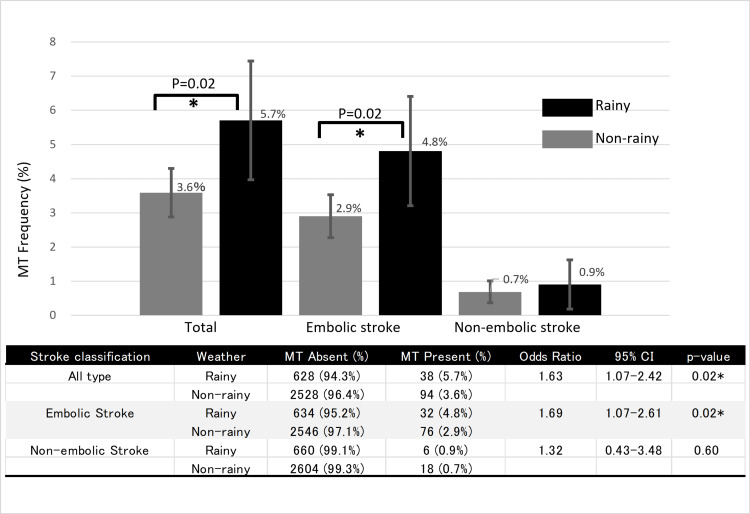
Comparison of mechanical thrombectomy (MT) frequency by rainy days versus non-rainy days and embolic stroke versus non-embolic stroke The bar graph shows the frequency of MT on rainy days (black bars) versus non-rainy days (gray bars) for total, embolic, and non-embolic strokes. Error bars represent 95% confidence intervals (CIs). Asterisks (*) indicate statistically significant differences (p < 0.05, Fisher's exact test) between rainy and non-rainy days. MT frequency was significantly higher on rainy days for total strokes (5.7% vs. 3.6%, p = 0.02) and embolic strokes (4.8% vs. 2.9%, p = 0.02) but not for non-embolic strokes (0.9% vs. 0.7%, p = 0.60). The table provides detailed statistics including MT absence and presence percentages, odds ratios, 95% confidence intervals, and p-values for each stroke classification. Percentages represent the proportion of days in each weather category on which at least one MT procedure was performed.

**Figure 3:**
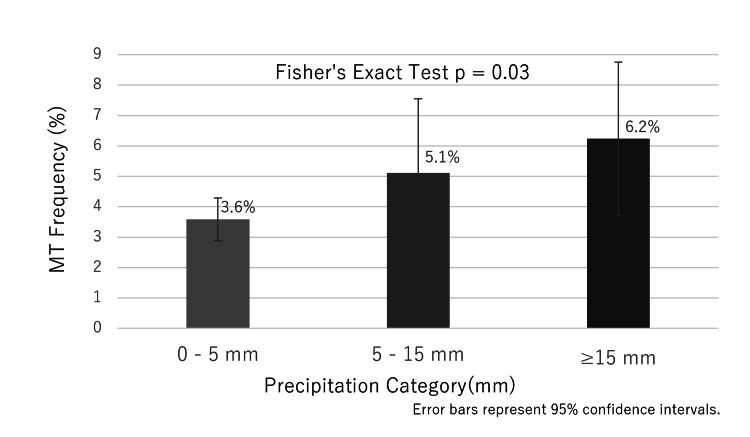
Mechanical thrombectomy (MT) occurrence frequency by precipitation category. The graph shows an increasing trend in MT occurrence frequency with higher precipitation levels (p = 0.03, Fisher's exact test). Error bars represent 95% confidence intervals. Percentages represent the proportion of days in each weather category on which at least one MT procedure was performed.

MT frequency was significantly higher on rainy days than on non-rainy days (5.7% vs. 3.6%; odds ratio (OR) 1.63, 95% confidence interval (CI): 1.07-2.42, p = 0.02) (Table 1). Percentages represent the proportion of days in each weather category on which at least one MT procedure was performed. To account for days with multiple MTs (one rainy and one non-rainy day each had two procedures), Poisson regression analysis was performed (incidence rate ratio 1.63, 95% CI: 1.07-2.42, p = 0.018). This association was driven by embolic strokes (p = 0.02), with no significant difference for non-embolic strokes (Fig. 2). MT frequency increased significantly with increased precipitation (p = 0.03) (Fig. 3). In a multivariate analysis adjusted for other meteorological factors, precipitation remained the only significant predictor of MT occurrence (OR 1.01 per mm increase, p = 0.015) (Table 1).

**Table 1:** Univariate and multivariate logistic regression analyses of weather conditions and MT occurrence Statistics: Fisher’s exact test (two-sided; exact p values) for proportions; logistic regression for OR (95% CI). "deviation": the difference between the day's meteorological value and mean of the preceding three days MT, mechanical thrombectomy; OR, odds ratio; AOR, adjusted odds ratio; CI, confidence interval; hPa, hectoPascals Multivariable model: adjusted for precipitation (primary exposure), minimum temperature, mean sea-level pressure, and maximum instantaneous wind speed; mean relative humidity was excluded due to strong correlation with precipitation. All variance inflation factors <2.5. The overall model did not show statistical significance (p = 0.14). Asterisks (*) indicate statistically significant differences (p < 0.05).

	Univariate analysis			Multivariate analysis		
Weather conditions	OR	95% CI	p-value	AOR	95% CI	p-value
Total precipitation (mm)	1.01	1.00-1.02	0.008*	1.01	1.00-1.02	0.015*
Maximum temperature (℃)	1.01	0.985-1.03	0.598	-	-	-
Maximum temperature deviation	0.967	0.914-1.02	0.243	-	-	-
Average temperature (℃)	1.01	0.987-1.03	0.457	-	-	-
Average temperature deviation	0.95	0.876-1.03	0.222	-	-	-
Minimum temperature (℃)	1.01	0.991-1.03	0.283	1.01	0.99-1.04	0.30
Minimum temperature deviation	0.994	0.92-1.07	0.878	-	-	-
Average humidity (%)	1.01	1.00-1.03	0.03*	-	-	-
Average humidity deviation	1.01	0.997-1.02	0.124	-	-	-
Average sea-level pressure (hPa)	0.99	0.965-1.02	0.463	1.01	0.98-1.05	0.45
Average pressure deviation	0.993	0.963-1.02	0.678	-	-	-
Minimum sea-level pressure (hPa)	0.993	0.969-1.02	0.577	-	-	-
Minimum pressure deviation	0.996	0.97-1.02	0.747	-	-	-
Maximum instantaneous wind speed (m/s)	1.02	0.973-1.07	0.411	1.02	0.97-1.08	0.41
Average wind speed (m/s)	1.06	0.912-1.23	0.453	-	-	-
Season (ref: summer)						
Autumn	1.01	0.627-1.63	0.963	-	-	-
Spring	0.911	0.558-1.49	0.708	-	-	-
Winter	0.868	0.528-1.43	0.577	-	-	-

Patient characteristics and treatment-related time metrics

Baseline patient characteristics, procedural details (e.g., first-pass effect and recanalization rates), and three-month clinical outcomes were comparable between rainy and non-rainy days (Table 2). EMS response time was similar, but the D2P time was shorter on rainy days than on non-rainy days (median: 101 vs. 131 min, p = 0.047). This difference was particularly pronounced during out-of-hours periods (99.5 vs. 142.0 min, p = 0.009) (Table 2).

**Table 2:** Comparison of mechanical thrombectomy (MT) cases on rainy versus non-rainy days Statistics: Mann–Whitney U (U statistic) for continuous variables; Fisher’s exact test for categorical variables. Values are median (IQR) or n (%). ASPECTS, Alberta Stroke Program early CT score; ATBI, atherothrombotic brain infarction; BA, basilar artery; CT, computed tomography; DWI, diffusion-weighted imaging; ESUS, embolic stroke of undetermined source; ICA, internal carotid artery; ICH, intracranial hemorrhage; MRI, magnetic resonance imaging; mRS, modified Rankin Scale; MT, mechanical thrombectomy; mTICI, modified thrombolysis in cerebral infarction; NIHSS, National Institutes of Health Stroke Scale; PC, posterior circulation; tPA, tissue plasminogen activator; VA, vertebral artery Asterisks (*) indicate statistically significant differences (p < 0.05). Tools/scales: NIHSS [9], mRS [10], ASPECTS/PC-ASPECTS [11-13], and mTICI [14]; all applied according to standard definitions. No license fees were required for non-commercial academic use.

	Rainy (N = 39)	Non-rainy (N = 95)	Test statistic (value)	p-value
Age	82 (67.5-86)	81 (74.0-86)	W = 1773	0.70
Sex (male)	19 (48.7%)	48 (50.5%)	N/A (Fisher’s exact)	1
Hypertension	24 (61.5%)	67 (70.5%)	N/A (Fisher’s exact)	0.31
Dyslipidemia	9 (23.1%)	34 (35.8%)	N/A (Fisher’s exact)	0.22
Diabetes mellitus	7 (17.9%)	23 (24.2%)	N/A (Fisher’s exact)	0.50
Baseline mRS 0-2 [10]	34 (87.2%)	79 (83.2%)	N/A (Fisher’s exact)	0.61
NIHSS [9]	17 (12–25.5)	19 (13.5–24)	W = 1796.5	0.79
MRI performed	19 (48.7%)	45 (47.4%)	N/A (Fisher’s exact)	1
tPA administered	18 (46.2%)	48 (50.5%)	N/A (Fisher’s exact)	0.71
Vascular location			N/A (Fisher’s exact)	0.18
ICA	13 (33.3%)	27 (28.4%)		
M1	11 (28.2%)	38 (40.0%)		
M2	8 (20.5%)	26 (27.4%)		
A1	0 (0%)	1 (1.1%)		
BA	5 (12.8%)	6 (6.3%)		
VA	2 (5.1%)	0 (0%)		
Others	1 (2.6%)	2 (2.1%)		
CT ASPECTS [11]	10 (9-10)	9 (8-10)	W = 938	0.18
DWI-ASPECTS [12]	7.5 (7.0-8.75)	7.5 (6.0-8.0)	W = 168.5	0.34
PC-ASPECTS [13]	8 (7.5-9)	8 (6.0-8)	W = 22.5	0.45
Stroke classification			N/A (Fisher’s exact)	0.18
Embolism (cardioembolic)	27 (69.2%)	57 (60.0%)	N/A (Fisher’s exact)	
Embolism (ESUS/others)	3 (7.7%)	19 (20.0%)	N/A (Fisher’s exact)	
Embolism (cancer)	2 (5.1%)	1 (1.1%)	N/A (Fisher’s exact)	
ATBI	6 (15.4%)	17 (17.9%)	N/A (Fisher’s exact)	
Dissection	1 (2.6%)	1 (1.1%)	N/A (Fisher’s exact)	
Emergency medical services response time (min)	32 (24.5-36.5)	29 (25.0-33.5)	W = 2081	0.26
Door-to-puncture time (min)	101 (71.5-132.5)	131 (85.0-167.0)	W = 1447	0.047*
- during out-of-hours (min)	99.5 (73.75-123.5)	142.0 (103.00-171.0)	W = 448	0.009*
- during in-hours (min)	126.0 (67.00-144.5)	117.5 (69.75-140.5)	W = 275	0.93
Time from call to treatment end (min)	214 (190.5-265.0)	244 (191.0-292.5)	W = 1628	0.27
Successful reperfusion (mTICI ≥2b) [14]	33 (84.6%)	85 (89.5%)	N/A (Fisher’s exact)	0.56
First-pass success (mTICI ≥2b) [14]	18 (46.2%)	40 (42.1%)	N/A (Fisher’s exact)	0.70
Symptomatic ICH	4 (10.3%)	5 (5.3%)	N/A (Fisher’s exact)	0.39
Asymptomatic ICH	10 (25.6%)	14 (14.7%)	N/A (Fisher’s exact)	0.39
Three-month mRS 0–2 [10]	16 (41.0%)	35 (36.8%)	N/A (Fisher’s exact)	0.99

## Discussion

This single-center, multi-year analysis demonstrates that daily precipitation is associated with higher utilization of MT and with shorter D2P times, particularly outside regular hours. The association appeared stronger for embolic etiologies and showed a dose-response pattern when precipitation was treated as a continuous exposure. Taken together, these findings position weather-specifically rainfall-as a pragmatic signal of service demand and workflow performance in real-world MT programs, rather than as proof of biological causation.

Interpretation of principal findings

First, the observation that rainy days coincide with higher MT utilization is concordant with prior reports suggesting that meteorological conditions can shape acute care pathways and transfer patterns [15,16]. In our data, the signal was most evident for embolic strokes and increased with greater precipitation, indicating that both clinical and operational/behavioral mechanisms may contribute. While it is tempting to infer a direct pathophysiologic trigger, the current design is better framed as a time-series association. The pattern could reflect modest short-term changes in stroke onset risk among embolic-prone patients and recognition and access factors that influence arrival within the MT window. While our study design cannot confirm behavioral mechanisms, plausible contributors include more time spent indoors with family (facilitating earlier symptom recognition), altered travel modes, and fewer competing activities on rainy days.

Second, D2P times were shorter on rainy days, with the largest differences during out-of-hours periods. Rather than a paradox, this likely reflects operational dynamics, for example, differences in on-call availability, reduced outpatient volumes, or more streamlined emergency throughput during inclement weather, each of which can compress pre-procedural intervals. Our observed efficiency gains on rainy days may reflect the inherent flexibility of optimized protocols like our institution's "Code AIS" system [17], which could be less susceptible to delays when regular hospital workflow is reduced. Our data do not establish which specific operational channels dominate, but they support viewing weather as a service signal that coincides with both higher demand and faster activation. Shortening D2P time is crucial for improving MT delivery, and various institutions have reported successful reduction strategies [17-20]. While prior work has focused on process design, no studies have evaluated weather as an operational signal, making our observations a novel angle that warrants further investigation alongside established pathways.

Thresholds versus dose response

Using a categorical definition (≥5 mm) yielded a clearer association than a lower threshold (≥1 mm). This does not necessarily imply a true biological threshold; it can also reflect nonlinearity in the exposure-response or improved signal-to-noise when separating minor drizzle from meaningful precipitation. Treating precipitation as a continuous exposure with potential nonlinearity is conceptually preferable and will be a focus of future work. Our observed dose-response using simpler models is consistent with this possibility.

Effect size and model performance

The estimated ~1% increase in MT odds per millimeter of precipitation is modest at the individual level but can translate into non-trivial operational impact at the system level when scaled across catchment populations, particularly during sustained rainy periods. The overall model’s modest explanatory power underscores that MT utilization is multifactorial, shaped by calendar effects, secular trends in protocols and eligibility, transfer practices, and stochastic variation. These features motivate multicenter time-series studies with standardized calendar/seasonal adjustments and pre-registered analytic plans.

Limitations

This study is limited by its single-center, retrospective design and potential misclassification of individual exposure at the time of stroke onset. Snowfall was uncommon, precluding inference on snow-related effects. Unmeasured or incompletely measured confounding, such as holiday schedules, secular changes in MT criteria or workflow, and varying transfer patterns, may influence results. These constraints support cautious interpretation and motivate external validation in diverse settings.

Clinical significance

Framing weather as a service signal has practical value. Centers may consider incorporating precipitation forecasts into demand forecasting, on-call staffing, and protocol readiness, especially for out-of-hours operations. The absence of detectable differences in short-term outcomes between rainy and non-rainy days in this cohort suggests that workflow efficiency gains did not translate into measurable discharge-level effects, but this warrants re-evaluation in larger samples. Overall, our findings are hypothesis-generating and operational in implication, aligning with prior literature that short-term meteorological shifts may influence embolic-prone populations and care-seeking patterns [8].

## Conclusions

Daily precipitation was associated with higher real-world utilization of mechanical thrombectomy and shorter D2P times, particularly outside regular hours. These findings support viewing weather, specifically rainfall, as a pragmatic service signal that coincides with both increased demand and more efficient activation, rather than as evidence of biological causation. The observed dose-response pattern using simpler models is consistent with potential nonlinearity, although formal modeling will be a focus of future work. Given the single-center, retrospective design and possible exposure misclassification, results should be interpreted cautiously. Incorporating precipitation forecasts into demand forecasting, on-call staffing, and protocol readiness may be operationally useful, especially for out-of-hours care. Multicenter, prospectively planned time-series studies are warranted to confirm generalizability, refine modeling of precipitation as a continuous exposure, and determine whether weather-informed operational strategies can sustainably improve timeliness of MT without increasing resource burden.
